# A Special Issue Introduction: The Intersection of Metacognition and Intelligence

**DOI:** 10.3390/jintelligence12090084

**Published:** 2024-08-31

**Authors:** Lisa K. Son, Hannah Hausman

**Affiliations:** 1 Department of Psychology, Barnard College, New York, NY 10027, USA; 2 Department of Psychology, University of California, Santa Cruz, CA 95064, USA

## 1. Introduction

What makes someone intelligent? Is it solving complicated math problems, understanding nuanced philosophical arguments, speaking many languages, finding practical solutions to real-world problems, or, more generally, being able to think and learn quickly? Although philosophers, religious figures, scientists, and educators have debated the definition and measurement of intelligence for hundreds of years, there is some generally agreed-upon notion that intelligence reflects one’s cognitive abilities, including our perceptual, problem-solving, reasoning, memory, language, and numerical skills (for a review, see Mackintosh 2011). We are less interested in defining what intelligence is compared to understanding what intelligence “gets us” as learners, teachers, employees, friends, partners, leaders, and members of society.

We distinguish between cognitive abilities, which are often measured by standardized intelligence tests (e.g., remembering words, creating analogies, noticing patterns, making deductions, mentally rotating shapes; Urbina 2011), and expressions of intelligence, sometimes referred to as successful intelligence (e.g., acing a chemistry test, making a wise investment decision, becoming an expert plumber; Sternberg 2018). Although the psychological concept of intelligence was originally framed in terms of explaining differences in performance across individuals, it is now well established that the same person, under different environments, can produce different intellectual results (Mueller and Dweck 1998), suggesting that cognitive abilities alone do not sufficiently explain how one’s intelligence manifests in various contexts. Even Wechsler, the creator of one of the most widely used intelligence tests today, acknowledged that “intelligent behavior, while often calling upon various acts of cognition, is not itself an aspect of cognition. It is not a kind of cognitive ability or faculty in the old sense of the term, certainly not in the sense that reasoning, memory, etc., have been so designated. Nor can it be equated or restricted to any combination of them”. Instead, he argues that “intelligence is an aspect of behavior; it has to do primarily with the appropriateness, effectiveness, and worthwhileness of what human beings do or want to do” (Wechsler 1975, p. 135).

What, then, determines the appropriateness, effectiveness, and worthwhileness of behavior? That is, what allows people to apply their cognitive abilities to succeed in their school, work, interpersonal relationships, finances, and health? And why do people do unintelligent things, even when they have the knowledge and skills to do better? We argue that the answer is metacognition—our thinking about our own thinking. This Special Issue is based on the idea that metacognition is a bridge between cognitive skills and expressions of intelligence throughout life. Cornoldi (2010) points out that “a theory of intelligence must explain the nature of intelligence expressions from failures to genius productions” (p. 265); we argue that invoking metacognition is necessary for any such explanations to be successful. Understanding the conditions under which metacognition is accurate or misleading is necessary for fully understanding when and why intelligent and unintelligent behaviors emerge.

Given our personal research interests in improving teaching and learning, this Special Issue is particularly focused on observable expressions of intelligence in an educational setting. Indeed, intelligence and education are deeply intertwined: One of the original uses of intelligence tests was to identify French children unprepared for primary education (Binet and Simon 1907). Furthermore, education is one of the most reliable ways known to improve intelligence (Ritchie and Tucker-Drob 2018). For better or worse, societies often equate prestigious schooling and academic achievement with intelligence. Articles in this Special Issue present new research relating metacognition to successful and unsuccessful expressions of intelligence in ways that are important for school, including learning and remembering during studying and test-taking.

## 2. Metacognition in Models of Intelligence

Metacognition can be broken down into monitoring and control. Metacognitive monitoring is our thinking about our own thinking, or our self-assessment of what we know, what we can do, how well we have learned something, how much we can remember, confidence we are on the right track with a solution, belief in an answer, estimates of how long a task will take, and more. Control is the self-regulated behavior that follows from monitoring, such as looking up information, asking questions, redoing a calculation, changing strategies, studying more, and blocking off time to work free of distractions (Nelson and Narens 1994). Several existing models of intelligence explicitly and implicitly involve metacognition, even though these models vary widely in their formulations of intelligence.

Metacognition has been shown to directly influence the cognitive processes associated with intelligence, including learning, memory, and problem solving (Kurtz et al. 1982; Cornoldi 2010; Hertzog and Robinson 2005). For example, working memory—i.e., the ability to actively maintain and manipulate information in one’s mind—is often conceptualized as a basic cognitive ability, or a core building block of intelligence (Cornoldi 2010; Shelton et al. 2010). Different people use different strategies when completing a working memory task, and people can improve with strategy instructions, suggesting that metacognitive monitoring and control influence working memory performance (McNamara and Scott 2001).

Researchers have also invoked metacognitive concepts such as self-reflection and self-regulation to explain how expressions of intelligence are not solely determined by cognitive abilities. Although not using the term metacognition, Wechsler (1975) argued that “intelligent behavior is not random but goal directed. It always has an intent and direction”, implicating an awareness of what one is doing and why (p. 137). By this definition, any expression of intelligence requires metacognitive monitoring and control to know where one is, how far one is from their goal, and the behaviors that bring one to that goal with the resources available (see also Hertzog and Robinson 2005). Similarly, Sternberg (2005) argues that intelligence involves people “recognizing their strengths and making the most of them, at the same time that they recognize their weaknesses and find ways to correct or compensate for them” (p. 104). It remains an open question what exactly the nature of the relationship is between metacognition and intelligence.

## 3. Problems with Viewing Metacognition as Intelligence

Very early on, some intelligence researchers have conceptualized metacognition as an indicator or type of intelligence. For example, Sternberg (1986) suggested in his early formal model that human intelligence might be divided into cognitive processes and metacognitive processes. While cognitive processes are responsible for how well people learn and use new information, metacognitive processes are equally important, guiding and receiving feedback from the cognitive processes. Similarly, Borkowski and Cavanaugh (1981) suggested that “metacognition stands as a potential conceptual candidate for inclusion in a general theory of intelligence and its accompanying assessment batteries” (p. 257).

One might be tempted to think that accurate metacognition is a good indicator of intelligence, and that poor metacognition reflects lower intelligence. After all, flawed self-assessment has negative consequences for education, health, and employment (Dunning et al. 2004), and people with the least knowledge or skill in a particular domain tend to be the most overconfident in that domain (Dunning et al. 2003). Yet overconfidence is not limited to those with the lowest intelligence as measured by knowledge and skills; it is a pervasive tendency that has been repeatedly demonstrated across decades, populations, and contexts (Fischhoff et al. 1977; Koriat et al. 1980; Loftus and Wagenaar 2014; Svenson 1981). Recently, stubborn overconfidence was found in tournament chess players, despite receiving years of feedback about their chess skills (Heck et al. 2024). Can we really consider all of the people in these examples of overconfidence, including proficient chess players, to be unintelligent?

We caution against interpreting metacognition as a type or indicator of intelligence because many factors outside individuals’ control or factors that we would not typically associate with intelligence can influence metacognitive accuracy. Inaccurate metacognitive monitoring can indicate nothing about intelligence. For example, those diagnosed with depression (Martin et al. 1984) and patients with obsessive–compulsive disorder (Dar et al. 2000) are not as overconfident as the general population. There may even be benefits to inaccurate metacognition. Cancer patients who believe they are healthier than other patients with similar prognoses have been shown to exhibit reduced distress (Helgeson and Taylor 1993). Inaccurate metacognitive monitoring has been suggested as a means by which people deal with difficult or uncertain situations (Choi and Son 2023), enabling them to sustain motivation and pursue a particular goal. For instance, people who overestimate their potential for success as new business owners are more likely to actually start businesses when compared with those who make more realistic predictions (Busenitz and Barney 1997).

Ultimately, metacognition is often associated with expressions of intelligence such as academic achievement, which we are interested in, but the prevalence of metacognitive errors among “intelligent people” or during intelligent behaviors suggests that metacognition and intelligence are related but distinct sets of cognitive processes. This Special Issue is based on the idea that the complexities of metacognitive monitoring and control must be investigated to understand the true nature of intelligence, especially with the aim of supporting students in expressing their intelligence in school and afterwards in their careers.

## 4. Accurate Metacognition Facilitates Expressions of Intelligence

We take the perspective that accurate metacognition facilitates expressions of intelligence but is not an expression of intelligence itself. One way to understand the role of metacognition in intelligence, especially in the context of learning and education, is to differentiate between intellectual potentiality and current intellectual ability (Ackerman 2018). Intellectual potentiality refers to domain-general cognitive abilities (e.g., working memory, reasoning, problem-solving), which are drawn on throughout learning and practice to develop one’s current intellectual ability (e.g., domain-specific knowledge and skills). Ackerman argues that it is intellectual ability rather than potentiality that primarily matters for people’s day-to-day expressions of intelligence in education, employment, and their personal lives.

Although metacognition is not referenced explicitly, Ackerman’s (1996, 2018) model identifies time and effort spent studying and practicing as the determinants of how intellectual potentiality becomes intellectual ability. Metacognitive monitoring and control are the cognitive processes that guide people’s decisions about what to practice, how to practice it, and for how long. Differences in manifestations of intellectual ability, or expressions of intelligence, as we call them, can therefore arise from differences in intellectual potentiality or differences in how time and effort were invested in practice to capitalize on this intellectual potentiality. For example, strong spatial reasoning skills such as the ability to mentally rotate and compare objects are a type of intellectual potentiality and predict performance in science, technology, engineering, and math (STEM) courses (Uttal and Cohen 2012). But spatial reasoning skills are not sufficient to do well in a STEM course; how and how much students study matters significantly for their learning and performance (Butler et al. 2014). Metacognitive monitoring and control processes drive such study decisions (Kornell and Finn 2016). In short, Ackerman’s model centers domain-specific knowledge and skills as the key determinants of expressions of intelligence; metacognition guides the practice needed to draw on one’s cognitive abilities to develop such knowledge and skills.

Cornoldi (2010) summarizes a model of intelligence that also differentiates between cognitive abilities and expressions of intelligence, which they refer to as the basic structure of intelligence and uses of intelligence, respectively. Unlike Ackerman, Cornoldi’s model explicitly refers to metacognition and centers on metacognition as the key to using the basic structure of intelligence for academic, professional, and personal achievement. The basic structure of intelligence in an educational setting includes processes that are more automatic (e.g., reading decoding, calculation skills) and processes that require much more conscious cognitive control (e.g., reading comprehension, spatial reasoning). Cornoldi (2010) suggests that “metacognition guides strategic and effective use of cognitive abilities” (p. 265), primarily influencing the highly controlled cognitive processes that make up the basic structure of intelligence. For example, reading comprehension is a more controlled process in the basic structure of intelligence, and metacognition influences how effectively students engage reading comprehension strategies in different contexts (e.g., searching memory for relevant prior knowledge, monitoring understanding to decide to reread a paragraph or look up a term, strategically reading for gaps in logic in a persuasive essay or the climax in a story). Thus, Cornoldi (2010) concludes that metacognition is “the most critical variable” that makes “basic intelligence applicable to real-life situations” (p. 274).

In short, Ackerman’s model of intelligence emphasizes the importance of domain-specific knowledge and skills, whereas Cornoldi’s model emphasizes highly controlled cognitive processes, yet both models include metacognition as a bridge between cognitive abilities and expressions of intelligence. Why is this bridge shaky at times?

## 5. Metacognition in Context

We know now, after more than three decades of metacognition research, that metacognition is not a single domain-general trait (e.g., Fitzgerald et al. 2017; but see Mazancieux et al. 2020) and cannot be interpreted independently of the context in which it is being examined. Metacognitive accuracy is heavily dependent upon what one is learning, how they are learning it, and the type of metacognition that researchers are assessing (e.g., Kelemen et al. 2000). Similarly, both Ackerman (1996, 2018) and Cornoldi (2010) point to social, emotional, motivational, and cultural factors that influence the expression of basic cognitive abilities as intelligence in everyday life.

Therefore, one major research priority for understanding and improving expressions of intelligence should be to elucidate the internal, personal factors and external, contextual factors that influence the accuracy of one’s metacognitive monitoring and the effectiveness of their metacognitive control in a given situation. We invited collaborators on this Special Issue to unpack a tiny part of this problem from their empirical perspective. Before elaborating on their contributions, we briefly review some interesting research that suggests that social and affective factors provide necessary context for explaining and reframing seemingly “unintelligent” metacognitive monitoring and control. This is especially important given the complex social–affective landscape that influences learning in school across all ages (Bliuc et al. 2017; Rozek et al. 2019; Schonert-Reichl 2019; Stadtfeld et al. 2019). Many of the contributions to the Special Issue relate to the question of social–affective influences on metacognition, too.

## 6. Social–Affective Influences on Metacognitive Monitoring Accuracy

Metacognitive monitoring is an individual’s insight into their knowledge and skills. And yet, metacognitive monitoring is not really personal at all; it can be directly affected by how one views themselves in the social context in which monitoring occurs. For example, Ehrlinger and Dunning (2003) had college student participants complete a scientific reasoning quiz and estimate their score on the quiz. Women’s estimates were lower than men’s estimates of their scores, even though women and men actually scored similarly on the quiz. Our metacognitive judgments, then, seem as though they are affected by others present in our shared context. Notably, students’ estimates of their quiz scores were significantly predicted by their self-view of their general scientific ability as compared with others, which was lower on average for women than men. In this case, social pressures that inform how we think we “ought” to do on a task can impair our ability to assess how we have actually done.

Students’ preconceived notions about their performance may be particularly problematic when students believe that a class or topic should be too difficult for them. For example, girls are socialized to incorrectly believe that they are not “math people” and report more math anxiety than boys (for a meta-analysis, see Else-Quest et al. 2010). The problem is that believing that something is going to be difficult can start a self-fulfilling cycle. When students went into a test believing that it would be relatively challenging, they reported that the test felt more difficult, took longer, and they did worse on the test, compared to when they went into the same test believing that it would be relatively easy (Critcher and Dunning 2009).

The breakdown in metacognitive monitoring stems from the fact that students’ beliefs about the task and their abilities influence how they interpret their subjective experiences while completing the task. Indeed, studies suggest that, compared to people who believe intelligence is more malleable, people who believe intelligence is more fixed tend to interpret experiences of disfluency during learning as a sign of a lack of learning, even if the disfluency is artificial and has nothing to do with actual learning (Miele and Molden 2010; Miele et al. 2011, 2013). This is particularly concerning for educators, since many of the most effective learning strategies feel more difficult in the moment than easier, but less effective strategies (Bjork and Bjork 2011; Soderstrom and Bjork 2015). Ironically, learning new information is a classic example of an expression of intelligence, and yet, people who are most concerned with demonstrating their intelligence may be the least likely to seek out and persist through the most beneficial learning opportunities, such as those that focus on promoting deeper understanding (Elliot et al. 1999; Greene and Miller 1996; Harackiewicz et al. 2000; Peng and Tullis 2020).

## 7. Social–Affective Influences on Metacognitive Control

Despite the general trend that monitoring influences control (e.g., Metcalfe 2009), there are many examples in which individuals’ control decisions seem “unintelligent”, not reflecting their relatively accurate monitoring. The notion that our assessment would guide our choices in behavior seems to be fairly straightforward, and yet, the literature is full of examples where the link between monitoring and control is broken, leading to suboptimal performances. For example, Sussan and Son (2014) found that high school students had the correct intentions, i.e., relatively accurate metacognitive monitoring. They expressed that they needed to study the unknown biology definitions more than those that were known. When it came to actual studying, though, none of the definitions were really studied at all. Similarly, Blasiman et al. (2017) found that college students knew that they would learn better by spacing out their studying over several days and weeks, and even intended to do so at the start of the semester; nevertheless, students ended up cramming their studying into the day or two before an exam. In these cases, one could easily write off their behavior as laziness, but the actual mechanism is not known. This is the complexity of metacognitive control—we can make assumptions about why people did or did not study, but they are merely assumptions. Recent research suggests that social and affective factors may help explain apparent disconnects between monitoring and control, especially in academic settings.

In terms of affect, seemingly poor metacognitive control can reflect a very intentional emotion-regulation strategy. For example, one common view holds that procrastination is a metacognitive monitoring problem in that people misjudge how long an assignment will take. Although true, there is also evidence that procrastination is an intentional coping strategy to temporarily avoid feelings of difficulty, discomfort, or failure and instead experience a positive mood (Pychyl and Sirois 2016).

Another example of the disconnect between monitoring and control is deliberate ignorance, which refers to the choice not to seek out potentially important information that one does not know. Researchers again suggest that one of the main reasons people do not try to learn information they do not know is for emotion-regulation purposes (Hertwig and Engel 2016). Many readers can probably relate to the deliberate ignorance of avoiding finding out their grade on an assignment or avoiding reading comments on a paper to put off the negative feelings that could come from critical but informative feedback. Indeed, students with test anxiety will avoid self-testing to avoid finding out how prepared or unprepared they are for an exam, even though self-testing is one of the most effective study strategies to enhance learning and academic performance (Liu et al. 2024). Thus, what seems like failures of metacognitive control based on researchers’ definitions of success may reflect effective, strategic behaviors just to achieve a different goal (e.g., emotion regulation rather than learning or performance).

In addition to seeking out information about one’s current level of knowledge and performance, another important metacognitive control strategy in school is to seek help externally, from, say, a teacher or a peer. Consider the situation where a student is confused about the lecture and intends to raise their hand to get clarification. Would every student raise their hand? There are varying assumptions that might be made about why students do not ask questions, why, say, they do not visit their teacher’s office hours, and why they might refrain from asking for feedback on a first draft. On the one hand, what seems like poor metacognitive control to not ask for help might actually be adaptive and reflect a student’s motivation to figure something out on their own. This avoidance of seeking help is present since childhood and can be thought of as the first sign of intelligence—the ability to seek more information internally (Jirout et al. 2024). Much of the literature on metacognitive control has focused on external information seeking for obvious reasons such as observability and measurability, but some of the most creative—that is, intelligent—solutions to filling the gaps in our knowledge might not yet exist in the external world.

Nevertheless, there are times we need to seek information and help externally, and failing to do so appears to reflect poor metacognitive control. There are powerful social factors that can explain the disconnect between monitoring and control in this case. One’s intersecting social identities undoubtedly affect metacognitive control choices, although this topic is greatly understudied. For certain minority groups especially, it is easy to imagine that a student might feel nervous about being the only one who is confused, about being different from their classmates, or even being “found out” as “not smart” by their teacher and peers. Indeed, people who fall high on the impostorism scale might be less likely to ask for feedback. First-generation students might feel anxiety about being found out that they do not understand as easily as others; East Asian students are less likely to say their opinions out loud; those of lower socioeconomic status seem to show a lower level of entitlement; and people who fell higher on the *Clance Impostorism Scale* (CIPS) were more hesitant to press a help button when solving math problems (Chen and Son 2024).

Such data suggest that even when one is aware that they do not fully understand the problem, the social context can make it unlikely for them to seek the necessary help to fill their knowledge gap. Studies suggest that underrepresented college students who participate in an intervention to increase their sense of social belonging at their university are more likely to engage in “intelligent” metacognitive control by using academic support services such as office hours and tutoring (Yeager et al. 2016; Walton and Cohen 2007). Similar results have been found with interventions that convey the message that one’s academic ability is not fixed and can improve with effort (Covarrubias et al. 2019).

In short, what may seem like unintelligent behaviors to researchers—such as not finding out important information, not starting work soon enough, or not asking for help—may actually be quite logical based on people’s social and emotional motivations. Unfortunately, these avoidance behaviors can be devastating to the learning process. As a result, the social context of schooling could dramatically undermine marginalized students’ abilities to effectively engage metacognitive control to develop and express their intelligence in academic settings.

## 8. Special Issue: The Intersection of Metacognition and Intelligence

The purpose of the Special Issue was to investigate the intricate relationship between metacognition and intelligence, particularly in terms of learning, memory, and test performance, given our interest in education. We organize our discussion of the articles in the special issues into three main categories: (1) evidence that metacognition guides expressions of intelligence; (2) insight into why the relationship between metacognition and intelligence is imperfect; and (3) open questions about how to target metacognition to increase intelligence, especially in the context of school. We hope that by providing a better understanding of the complexity of metacognition and the way it influences intelligence, this Special Issue will help change stereotypical and inflexible views of an “intelligent” or “unintelligent” person.

### 8.1. Metacognition Guides Expressions of Intelligence

Survey research by Coane et al. (2023) found that—like the frameworks for intelligence summarized above—members of the general public believe that intelligence is multifaceted and reflects the accumulation of knowledge (Ackerman 1996, 2018). Their beliefs also echo claims in the literature that intelligence is more than just having knowledge and skills; it involves effectively applying one’s knowledge and skills to learn new information and solve new problems, including in creative, non-traditional ways.

Other articles in the Special Issue provide insight into how metacognition can bridge merely having knowledge and using it effectively to express one’s intelligence. One notable and often frustrating feature of knowledge is that just because we possess it does not guarantee we can remember it when we want or need to (Bjork and Bjork 1992). Experiences such as tip-of-the-tongue (TOT) states, déjà vu, and déjà entendu are ways in which metacognition gives individuals insight into whether the relevant knowledge is in their memory. Lee et al. (2023) found that even when people could not recall information themselves, they were more likely to be able to recognize it when they were in a TOT state versus not. McNeely-White and Cleary (2023) found that such metacognitive experiences—feelings of knowing something even if it is currently unrecallable—make people curious and motivate them to learn more. When participants were experiencing déjà vu and déjà entendu, they spent more time trying to remember why and were more willing to spend limited resources to figure it out. Thus, metacognitive monitoring is critical for expressions of intelligence because it guides effective control of cognitive processes such as memory retrieval and information seeking (Cornoldi 2010).

In some circumstances, though, it may be more intelligent to stop trying to think of an answer or solve a problem rather than persist. Here, too, metacognition can help. Arslan and Finn (2023) observed in a study of students’ problem-solving behaviors on digital math tests that students would sometimes answer math questions incorrectly by rapidly guessing an answer. Personalized “nudges” or reminders to put in effort on these problems successfully increased how long students spent trying to solve problems, but their test scores did not improve as a result. The results indicated that rapid guessing is not a sign of poor metacognitive control but rather a reflection of students’ strategic behavior based on relatively accurate insight into which problems they did not know how to solve, and more so than what the testing software could infer.

Taken together, these studies suggest a more nuanced view of what is considered intelligent behavior is needed. Overall, persistence and grit are important predictors of academic achievement (Lam and Zhou 2019), but metacognition appears to be key for determining at a micro-level (e.g., a specific question or problem) whether persistence is intelligence or fruitless stubbornness for that learner in that moment (Nelson and Leonesio 1988).

### 8.2. An Imperfect Relationship between Metacognition and Intelligence

Although many of the articles in the Special Issue reveal an association between metacognition and intelligence, the two processes are not in perfect correspondence. The articles in this category reveal examples of inaccurate metacognition coupled with high levels of performance and accurate metacognition but low levels of performance.

The link between metacognition and intelligence is imperfect, in part because metacognitive monitoring is an inherently noisy process. People do not have direct access to the contents and strength of their memories but have to infer them based on cues. Metacognitive monitoring judgments will be inaccurate when the cues used are not valid indicators of what one actually knows (Koriat 1997). Because of the inferential nature of metacognition, high levels of performance can be associated with relatively inaccurate metacognition. Serra and Shanks (2023) found that participants judged their ability to remember specific facts on the basis of domain familiarity or knowledge, which is a relevant but imperfect cue. Participants’ confidence and memory performance were highest for facts from domains they knew the most about in general, but participants were also the most overconfident in their memory in these domains.

Similarly, Little (2023) compared different types of multiple-choice test questions and found that while people perform equally well on the questions that include the option of “none of the above” (NOTA) and non-NOTA questions, people feel much less confident in the former. Participants’ confidence was partially influenced by their overarching belief that NOTA questions are more difficult and “trickier” in general, not just their experience answering the specific NOTA questions on the test.

If metacognition guides the development and expression of intelligence, how could articles in the Special Issue find relatively high levels of performance accompanied by relatively low metacognitive monitoring accuracy? Given that metacognitive monitoring influences control, inaccurate monitoring will likely have a smaller negative impact on intelligence when people have relatively little control over the task, the environment, or their performance. Serra and Shanks (2023) did not give participants a chance to review material after making metacognitive judgments but before taking the test. Similarly, there is very little that participants could change about how they controlled their cognition while answering NOTA versus non-NOTA questions (Little 2023). Unlike with a short-answer or essay test, slight changes in the multiple-choice test question format do not allow people to control how much information they recall, whether they provide more factual details or conceptual arguments, the organization of their answer, etc. As a result, features of the tasks affected how people judged their learning or performance but did not provide people an opportunity to act on them, leading to a decoupling of metacognition and test-based measures of intelligence.

One area in which learners have significant control is their studying. It is here that we would expect inaccurate metacognition to have a much more detrimental effect on the development and expression of intelligence. In the current issue, Do and Thomas (2023) provide more evidence that people are often not aware of how different study strategies have affected their learning (Bjork et al. 2013; Soderstrom and Bjork 2015). Participants learned more when they practiced similar concepts interleaved, or in an intermixed order, compared to when practice was blocked by concepts, but participants believed they learned more from blocked practice. In this experiment, the study strategy was experimentally manipulated. If people were given the choice of how to study, inaccurate monitoring could lead to ineffective strategy use and suboptimal learning (Tauber et al. 2013).

The previous papers in this section demonstrate examples of low metacognitive accuracy yet high levels of performance. Conversely, Choi and Son (2023) show that having accurate monitoring is not enough to ensure intelligent reasoning or decision-making. The hot-hand heuristic refers to when people believe a string of successes is more likely to lead to more successes than failures, even when the odds are random. In this study, some people were “faithful” hot-hand believers. Curiously, the higher their metacognitive accuracy, the more likely they were to be affected by having a “hot hand” in a gambling task. Their gambling decisions may have been driven by something other than metacognitive awareness of the logically correct choice. It might have been more exciting or interesting for those participants to see if they could keep the winning streak going. Someone might be engaging in unintelligent behavior but know better because they have other motivations, which makes it appear that metacognition and intelligence are less related than they actually are.

### 8.3. Targeting Metacognition to Improve Intelligence

Even though metacognition and intelligence do not always go hand in hand, on balance, we believe that metacognition would be a good target for improving intelligence, especially in academic settings (see also Cornoldi 2010). Educators should undoubtedly focus on teaching domain-specific content and skills, but Karaca et al.’s (2023) findings suggest that improving metacognition is critical for students to capitalize on these learning opportunities to develop and express their intelligence. University students predicted their grades on four exams in a course and rated their confidence in the accuracy of their predictions. The lowest performing students overestimated their exam scores the most, were quite confident in their inflated exam score predictions, and remained confident in them across all four exams, even though they were receiving objective evidence after each new exam that they were not learning as much as they had thought. That is, students never became aware of the fact that they were not learning. This goes against the Memory-for-Past-Test findings, where once an individual has taken a test and performed poorly, their subsequent metacognitive judgments tend to fall, as they should (Finn and Metcalfe 2008). Future research is needed to understand why people use their past test performance to inform their predictions of future test performance in the lab but not in real courses (Foster et al. 2017). Karaca and colleagues’ findings suggest this discrepancy may have something to do with awareness.

It is not necessarily a sign of unintelligence for students to perform poorly on an exam because the material has not been well-learned yet. To us, the true sign of intelligence would be that students can recognize that their performance did not match their expectations and change how they monitor and control their learning going forward. Researchers have identified myriad strategies that can help students more accurately monitor their current understanding of specific course material, such as having students judge their learning only after waiting some period of time after a lecture, reading assignment, or study session and then trying to remember or explain the material from memory. As a result, students engage in more effective self-regulated study decisions and learn the information better in the long run (Hausman et al. 2021). Based on Karaca and colleagues’ results, we wonder if these strategies are enough to meaningfully improve academic achievement. Students may also need support with bigger-picture metacognitive reflection to become aware of whether their current approach to learning and classes is working as intended, moving them closer to their academic goals. This is an important area for future research because exam wrappers—i.e., post-exam reflection assignments—have shown only modest and inconsistent benefits for students’ metacognition and course performance (Hodges et al. 2020; Soicher and Gurung 2017).

The research presented by O’Leary and Fletcher (2024) provides converging evidence that students do not automatically engage in metacognitive reflection and need help doing so to truly be “intelligent”. Explicitly prompting people to reflect on the counterevidence that was presented led people to update their pre-existing beliefs more than reading the counterevidence alone (numerically, if not statistically significantly). In the real world, belief updating in light of new evidence is perhaps the epitome of the learning and intelligent behavior we hope to cultivate in school. This finding is in line with the literature on other metacognitive biases. People tend to be overconfident in their answers to questions and, after finding out an answer was wrong, will learn the correct answer and feel like they knew it all along (the hindsight bias). When asked to think about the opposing outcome, people are less likely to be overconfident in their answer or to have known the answer prior to learning it (Koriat et al. 1980). It seems that one type of metacognitive process that is especially important for intelligence is a flexible metacognitive reflection about what you know, how you know it, and if you need to update, correct, or learn more. Getting people to update their knowledge and beliefs may require more than just prompts to reflect, though. Recent data have shown that when people disagree with a statement, they rate it as more of an opinion than a fact (Brotherton and Son 2021).

Given the fallible nature of metacognitive monitoring, one might ask whether it truly makes sense to try to teach people to engage in more metacognitive reflection. With increasingly sophisticated educational software and artificial intelligence, it is possible that the best approach to improving education would be to have algorithms and technology dictate what students learn, in what order, how long they spend on problems, etc. Hays and colleagues show that computer-based educational training platforms, including online learning, can counteract biases that occur in human-driven learning. Such research continues to ask the Turing-test-like question from the perspective of awareness: Who is the better ‘teacher’: us or the machine? If it’s the machine, then, shockingly, metacognition and awareness may not be key to developing and expressing intelligence. We think this answer is unlikely since there are examples in which humans learn more or perform better when they are given control rather than have to follow the normatively good decisions of a computer, suggesting that intelligence requires the meta in metacognition (e.g., Arslan and Finn 2023; Nelson and Narens 1994; Son 2010; Tullis and Benjamin 2011).

A part of why we believe that we cannot improve human intelligence without awareness is that learning occurs in a social context in the real world. Yet, there is an enormous amount of data showing that an individual’s performance on a test can be affected by others, even when no verbal exchanges take place. The classic literature on stereotype threat was the first to investigate the possibility that the descriptions of others, including gender and race, are enough to bias someone’s “intelligence”, as manifested by test performance (Spencer et al. 2016). A lifetime of social observation, experiences, and messaging creates cultural values and norms that influence how people control their cognitive processes (Cornoldi 2010). In the current issue, Ackerman et al. (2023) show that individuals from varying cultures exhibit different levels of monitoring accuracy, effort regulation, and, subsequently, success. This paper highlights the idea that intelligence is related to metacognitive processes, but can also be swayed by contextual factors, such as one’s culture or upbringing.

One of the most important areas for future research is to explore social-cultural influences on metacognition, measures of intelligence, and the influence of metacognition on intelligence. Indeed, in almost all of the literature on metacognitive monitoring and control, processes have been examined under conditions in which participants learn individually (e.g., Tsalas et al. 2017; Miller and Geraci 2011). Very few studies have looked at metacognition in social situations. For instance, one study suggested that an individual’s metacognitive processes could also involve the monitoring and control processes of the larger environment (Chiu and Kuo 2009). On the other hand, others, like Carruthers (2009), explicitly set the social component apart from metacognition, referring to making inferences about others’ cognitive processes as “mind reading”. Future research should consider the role of metacognition for the self and others in collaborative intelligence—i.e., the ability to transform and apply knowledge to answer questions and solve problems with others. The intelligent performance of a group reflects more than just the intelligence of its members; it is also influenced by the group members’ social sensitivity (Woolley et al. 2010).

If intelligence is about real-world, everyday achievement, then perhaps one of the most important expressions of intelligence is not how people use their cognitive abilities to solve problems in isolation, but how they work in concert with others to achieve a shared goal. Understanding how individuals use metacognition to monitor their own thinking, the thinking of their collaborators, and control their own cognition will provide rich insights into how to not only improve the intelligence of individuals but also ensure their intelligence is put to good use to collaboratively tackle societal issues.

## 9. Conclusions

It was Einstein who said that “the measure of intelligence is the ability to change”. We agree with this statement, but first propose that there needs to be an advanced understanding of the factors that can lead to existing manifestations (performance, problem solving, decision-making, etc.) of intelligence. Metacognition, a potentially very personal reflective process, seems to be a crucial component of how people can develop and demonstrate incredible feats of intelligence. But, at the same time, intelligence can appear unstable, both across situations within one individual as well as across individuals and groups in the same situation. The papers in this Special Issue reflect some of that instability, and, we hope, will motivate continued research at the intersection of metacognition and intelligence, eventually indicating that the highest form of intelligence is, perhaps, instability.

## Data Availability

Not applicable.
